# Fast Fabrication Nanopores on a PMMA Membrane by a Local High Electric Field Controlled Breakdown

**DOI:** 10.3390/s24072109

**Published:** 2024-03-26

**Authors:** Shaoxi Fang, Delin Zeng, Shixuan He, Yadong Li, Zichen Pang, Yunjiao Wang, Liyuan Liang, Ting Weng, Wanyi Xie, Deqiang Wang

**Affiliations:** 1 Chongqing Key Laboratory of Multi-Scale Manufacturing Technology, Chongqing Institute of Green and Intelligent Technology, Chinese Academy of Sciences, Chongqing 400714, China; fangshaoxi@cigit.ac.cn (S.F.); heshixuan@cigit.ac.cn (S.H.); wangyunjiao@cigit.ac.cn (Y.W.); liangliyuan@cigit.ac.cn (L.L.); wengting@cigit.ac.cn (T.W.); 2 Chongqing School, University of Chinese Academy of Sciences, Chongqing 400714, China; 3 School of Optoelectronic Engineering, Chongqing University of Posts and Telecommunications, Chongqing 400065, China; delin_zeng@163.com (D.Z.); liyadong@cigit.ac.cn (Y.L.); pangzichen@cigit.ac.cn (Z.P.)

**Keywords:** PMMA, nanopore, controlled breakdown, local high electric field, low noise

## Abstract

The sensitivity and accuracy of nanopore sensors are severely hindered by the high noise associated with solid-state nanopores. To mitigate this issue, the deposition of organic polymer materials onto silicon nitride ($SiN_{x}$) membranes has been effective in obtaining low-noise measurements. Nonetheless, the fabrication of nanopores sub-10 nm on thin polymer membranes remains a significant challenge. This work proposes a method for fabricating nanopores on polymethyl methacrylate (PMMA) membrane by the local high electrical field controlled breakdown, exploring the impact of voltage and current on the breakdown of PMMA membranes and discussing the mechanism underlying the breakdown voltage and current during the formation of nanopores. By improving the electric field application method, transient high electric fields that are one–seven times higher than the breakdown electric field can be utilized to fabricate nanopores. A comparative analysis was performed on the current noise levels of nanopores in PMMA-$SiN_{x}$ composite membranes and $SiN_{x}$ nanopores with a 5 nm diameter. The results demonstrated that the fast fabrication of nanopores on PMMA-$SiN_{x}$ membranes exhibited reduced current noise compared to $SiN_{x}$ nanopores. This finding provides evidence supporting the feasibility of utilizing this technology for efficiently fabricating low-noise nanopores on polymer composite membranes.

## 1. Introduction

Nanopores have been proven to be an important tool for detecting biomolecules and their conformations [1], including nucleic acids [2,3,4,5], proteins [6,7], and polymers [8] that play a vital role in life and medical treatment. Because of their controllable pore size, environmental adaptability, chemical stability, scalability, and integration capabilities [9], solid-state nanopores are considered an effective substitute for biological nanopores [10,11]. However, the significant challenge of high-noise interference in solid-state nanopores remains the most serious obstacle limiting their use in DNA sequencing [12,13].

The interaction between the ionic solution and the nanopore wall [14], as well as the membrane surface, are the primary contributors to nanopore noise [15,16]. This noise is directly influenced by the material and structure of the membrane [17,18,19], including electron mobility, dielectric constant, contact area, and thickness. Using a low dielectric constant and reducing the suspended area are two effective methods for reducing noise in nanopores. Balan et al. proposed a high-frequency noise reduction structure aimed at reducing the capacitance of silicon nitride membrane, by adding thick insulating layers to the chip surface [20]. The incorporation of silicon dioxide layers effectively decreased the capacitance of the silicon nitride nanopore to a range of 1–5 pF, enabling high-frequency acquisition at 1 MHz. Pitchford et al. implemented a similar approach by substituting the cover layer of the silicon nitride ($SiN_{x}$) membrane with a polymer material [21]. This modification not only achieved low-noise acquisition but also mitigated optical interference. These studies demonstrate that constraining the contact area between the silicon nitride membrane and the ionic solution through the cover layer, while reducing the capacitance, serves as an effective strategy for noise reduction [22]. Furthermore, it has been demonstrated that low dielectric constant materials such as silicon dioxide and polymer are excellent choices for fabricating solid-state nanopores [23,24]. Junseo et al. proposed a novel method capable of creating sub-10 nm conical nanopores in a freestanding polymer membrane [25], which was validated through DNA translocation experiments. In this fabrication process, nanoimprint lithography (NIL) is utilized to produce the initial nanopores, followed by a polymer reflow process to reduce the pore diameter with fine control. These nanopores exhibit superior noise performance; however, they enhance spatial resolution and further reduce the pore diameter pose significant challenges [26].

Polymethyl methacrylate (PMMA) is an organic polymer extensively utilized in medical polymer materials and electronic devices due to its excellent chemical stability, superior dielectric properties, and electrical insulation capabilities [27,28]. This article focuses on PMMA as a representative material to investigate the fabrication of nanopores through dielectric breakdown on organic polymer-$SiN_{x}$ composite membranes.The dielectric breakdown method for solid-state nanopore fabrication was initially proposed by the Kwork group in 2012 and achieved the in situ fabrication of silicon nitride nanopores in 2014. This method allowed for the production of nanopores as small as 2 nm, significantly reducing the cost and complexity of nanopore processing [29,30,31,32,33]. However, in the fabrication process of this method, there is extensive contact between the electrolyte solution and the membrane, resulting in uncertain positioning of the nanopores and the potential formation of multiple nanopores simultaneously. To address these issues and improve control over nanopore formation, the technique of local electric field dielectric breakdown technology was developed [34,35,36,37].

This article proposes the application of the transient high electric field dielectric breakdown method to fabricate nanopores on PMMA-$SiN_{x}$ composite membranes, building upon the research group’s prior work [34,35]. Initially, conventional and cost-effective spin coating techniques were employed to fabricate PMMA membranes on silicon substrates coated with an Au electrode layer. The study investigates the impact of voltage and current on the breakdown of the PMMA membrane. Additionally, the paper delves into the mechanism behind the breakdown of voltage and current during nanopore formation. Finally, a comparison and analysis of the current noise between nanopores in PMMA-$SiN_{x}$ composite membranes and $SiN_{x}$ nanopores fabricated through local dielectric breakdown are conducted, demonstrating the method’s viability for fabricating low-noise composite membrane nanopores.

## 2. Materials and Methods

Lithium chloride (LiCl, 99.9%), polymethyl methacrylate (PMMA, $M_{W}$∼15,000), Ethylenediaminetetraacetic acid disodium salt (EDTA-Na_2_2H_2_O), and Trizma base (Tris, 99%) were purchased from Sigma-Aldrich (St. Louis, MO, USA). Silver wire (99.99%) was purchased from WPI (Sarasota, FL, USA). Sodium hypochlorite and ethyl lactate were purchased from KESHI (Chengdu, China).

Preparation of electrolyte: The 1 M LiCl electrolyte solutions were buffered with 10 mM Tris and 1 mM EDTA and adjusted to a pH of 8.0. The deionized water with an electrical resistance of >18.2 MΩ was supplied by a Millipore Milli-Q water purification system (Billerica, MA, USA).

Ag/AgCl electrode preparation: The silver wire is immersed in sodium hypochlorite overnight to make Ag/AgCl electrodes. Then, the micropipette is filled with 1 M LiCl solution and is connected to the source meter (Keithley 2450, Tektronix, Beaverton, OR, USA) through the Ag/AgCl electrodes.

Preparation of PMMA membrane: Dissolve the PMMA powder in ethyl lactate to create a PMMA solution with concentrations (mass ratio) of 2%, 3%, 4%, and 5%, respectively. Silicon wafers (P, <100>) or gold-plated silicon substrates are used as substrates. Firstly, the substrate is immersed in deionized water and alcohol, sonicate for 10 min and baked on a hot plate at 120 °C. Then, a coater is utilized (AC200-SE, Schwan Technology, Jiangying, China) to fabricate PMMA membranes at various concentrations and rotational speeds.

$SiN_{x}$ membranes (NT001Y, Norcada, Edmonton, AB, Canada) with 10 μm × 10 μm window size and 20 nm thickness were used in all experiments.

## 3. Results

### 3.1. Preparation of PMMA Membrane

In comparison to silicon, silicon nitride, graphene, and molybdenum disulfide, PMMA offers several advantages in the fabrication of nanopores.These include its simplicity in being fabricated into various shapes and structures, which makes it highly suitable for rapid prototyping and manufacturing processes [38]. Moreover, PMMA exhibits a wide range of physicochemical properties, surface-modification protocols, and minimal noise, allowing for versatile applications [27,28]. However, it is important to note that fabricating nanopores in a polymer membrane presents challenges due to the need for high aspect ratio molding. This is because reducing the pore size cannot be accompanied by the same reduction in the thickness of the polymer membrane. Maintaining the mechanical strength of the polymer membrane is crucial during this process [39]. The thinnest reported polymer membrane for nanopore fabrication is 1 μm [25]. To enhance the spatial resolution of polymer nanopores, this article employs a composite membrane consisting of a PMMA membrane and an $SiN_{x}$ membrane with nanoscale thickness for nanopore fabrication.

The engineering value of the breakdown electric field ($E_{bd}$) of PMMA membrane is 18–22 MV/m [40], and the breakdown voltage can be obtained according to Equation (1):

(1) $$E_{bd}=\frac{U}{t}\left(\mathrm{MV}/m\right)$$

where $E_{bd}$ is the breakdown electric field, *U* is the applied voltage, and *t* is the membrane thickness, which plays a crucial role in determining the fabrication electric field. In Figure S1 (Supporting Materials), a coater is utilized (AC200-SE, Schwan Technology, Jiangying, China) to fabricate PMMA membranes at various concentrations and rotational speeds. To ensure clear observation of the dielectric breakdown phenomenon (nanopore fabrication stage in the process of PMMA nanopore fabrication) when the pipette tip is in contact with the membrane, it is necessary to maintain the source voltage above 2 V. Consequently, in the experiment, a PMMA membrane with a thickness of 100 nm (In Figure S2, Supporting Materials) produced at a 5% concentration and a rotational speed of 3000 rpm was chosen as the material for subsequent experiments. Its surface was assessed using AFM (Dimension Edge, Bruker, DE, USA), confirming that its surface roughness was less than 5 nm.

### 3.2. Platform for PMMA Nanopore Fabrication

**Figure 1:**
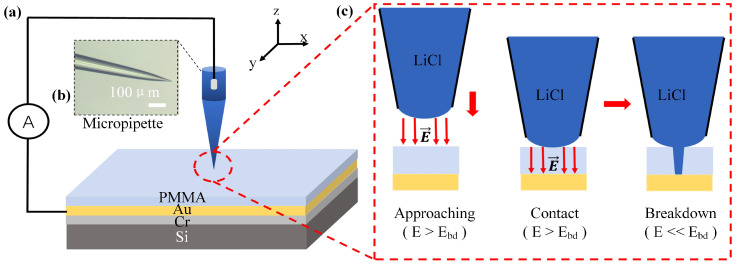
The method of fabrication of a PMMA nanopore by a local high electric field breakdown. (**a**) Schematic diagram of the experiment device. The PMMA membrane is coated on the sandwich substrate (gold, chromium, and silicon). The fabrication electric field between the micropipette and gold is controlled by the positioning platform and the source meter. (**b**) The enlarged view of the micropipette. (**c**) The PMMA nanopore fabrication process by the local high electric field between the pipette tip and the gold electrode layer.

Figure 1 illustrates the method of fabrication PMMA and $SiN_{x}$ nanopores through local high electric field breakdown [34]. To simplify the fabrication process and the characterization of PMMA nanopores, the fabrication of PMMA nanopores is directly conducted on sandwich substrates comprising gold, chromium, and silicon. A chromium (Cr) layer and a gold (Au) layer are successively sprayed onto the silicon substrate using a spray coating process to create the sandwich substrates. Initially, the chromium layer helps prevent peeling or detachment during subsequent processing steps, while the gold layer offers excellent conductivity and stability, making it suitable for use as an electrode during the nanopore fabrication process, where an electric field is applied. Subsequently, the PMMA membrane is spin-coated onto the substrate and dried using a homogenizer. The micropipette, pulled from borosilicate capillary tubes (BF100-78-10, Sutter, Novato, CA, USA) using a Sutter P-2000 micropipette puller, is displayed in Figure 1b. By adjusting the instrument parameters detailed in Table S1 (Supporting Materials), pipette tips of varying sizes can be produced.

The micropipette is filled with 1 M LiCl solution and connected to the source meter (Keithley 2450, Tektronix, Beaverton, OR, USA) via the Ag/AgCl electrodes. After applying a high voltage between the tip and the Au electrode layer, the pipette tip approaches the membrane. Upon contact with the PMMA membrane, a high electric field is formed (as shown in Figure 1c), leading to the breakdown of the PMMA membrane and the formation of a nanopore. The electric field strength, stimulation area, and position of the high electric field are controlled by the voltage, tip diameter, and the three-axis high dynamics nanopositioning system (P363 PicoCube, PI, Karlsruhe, Germany) along with a controller (E-536, PI, Karlsruhe, Germany).

### 3.3. The Process of PMMA Nanopore Fabrication

The influence of electric field strength on the breakdown process using controlled dielectric breakdown can be divided into three types:

(1) Far below breakdown field (*E* ≪ $E_{bd}$): The applied electric field strength is much lower than the material’s breakdown field ($E_{bd}$), so dielectric breakdown does not occur, and the solid-state nanopore cannot be formed.

(2) Gradually approaching breakdown field (*E* ≤ $E_{bd}$): When the applied electric field gradually approaches the material’s breakdown field strength, with the accumulation of time, it leads to structural changes in the material, eventually forming a solid-state nanopore.

(3) Above breakdown field (*E* > $E_{bd}$): When the applied electric field strength exceeds the material’s breakdown field strength, solid-state nanopores are formed instantly. In this case, the electric field is powerful enough to immediately trigger structural changes in the material, leading to the formation of a nanopore.

In traditional dielectric breakdown methods, an uncontrolled electric field higher than the breakdown field would directly damage the membrane. However, in the author’s previous research [34,35], precise control of the electric field’s action time and area was achieved through the nanopositioning system, enabling localized fabrication of solid-state nanopores. This method of local dielectric breakdown will be applied to the fabricate of PMMA nanopore in this study.

**Figure 2:**
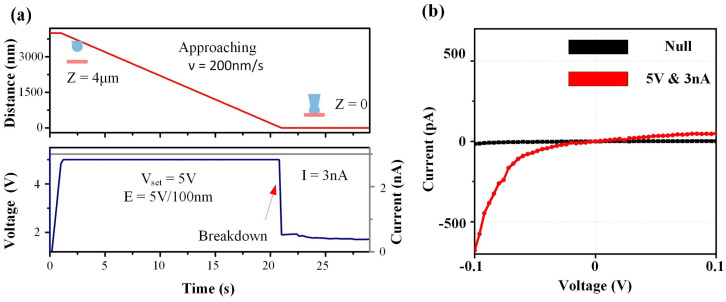
The process of PMMA nanopore fabrication via local high electric field controlled breakdown. (**a**) The output voltage and power will gradually rise to the pre-set voltage (5 V) when the micropipette is suspended 4 μm above the membrane. As the pipette tip approaches the membrane at a speed of 200 nm/s, when the tip contacts the membrane, it will form a PMMA nanopore due to the high electric field. Simultaneously, the load of the current source becomes the nanopore resistance, and the electric field is small. (**b**) The conductance of PMMA membrane before and after fabrication by the local high electric field breakdown.

In Figure 2a, the fabrication process is divided into four stages: constant current source pressurization, pipette tip approching, nanopore fabrication, and nanopore expansion.

(1) Constant current source pressurization stage: During the fabrication of PMMA nanopores process, an electric field greater than the breakdown electric field needs to be applied to fabricate the nanopores. According to Equation (1), the breakdown electric field for a 100 nm thick PMMA membrane ranges from 1.8 V to 2.2 V. It is necessary to select a voltage of 2.2 V or above to provide a local ultra-high electric field for fast fabrication of PMMA nanopores. As shown in Figure 2a, a voltage of 5 V was set for breakdown.

In addition, previous studies have shown that the current of the constant current source affects the speed of voltage application and the process of nanopore expansion. When the constant current source is too small (less than 0.5 nA), the rate of increase in applied voltage is slow, which significantly impacts the efficiency of nanopore fabrication. On the other hand, when the constant current source is too large (greater than 50 nA), the expansion speed is faster, resulting in a high flow rate of electroosmotic flow. This can affect the target pore size and processing accuracy of nanopore fabrication. Therefore, in this experiment, the range of the constant current source is controlled ranging from 1 nA to 50 nA. A constant current of 3 nA was selected, as shown in Figure 2.

(2) Pipette tip approching stage: Utilizing a piezoelectric driving platform to control the glass Pipette tip as it approaches the PMMA membrane at a speed of 200 nm/ms while detecting real-time changes in feedback voltage.

(3) Nanopore Fabrication stage: When the glass micropipette tip contacts the PMMA film, the PMMA nanopores quickly forms, and the feedback voltage decreases rapidly.

(4) Nanopore expansion stage: Following the formation of the PMMA nanopore, a low-noise, high-speed current detection circuit is employed to test the IV curve of the nanopore and calculate the pore size of the PMMA nanopore. The results in Figure 2b indicate the successful fabrication of a nanopore on the PMMA membrane. During this stage, the IV curve test is used to determine the initial pore size of the nanopore formed instantaneously at the fabrication voltage. Subsequently, by continuously applying a constant current source, the diameter of the nanopore gradually expands. The larger the applied current, the faster the expansion rate, until reaching the desired target pore size for fabrication.

### 3.4. Voltage Effect for PMMA Nanopore Fabrication

The prerequisite for the fast fabrication of solid-state nanopores using the controllable local dielectric breakdown method is that the applied electric field of the system is higher than the breakdown electric field. Therefore, applying an electric field is is a fundamental aspect of the precise fabrication of solid-state nanopores. Further research is required to explore the impact of applying an electric field on nanopore fabrication.

**Figure 3:**
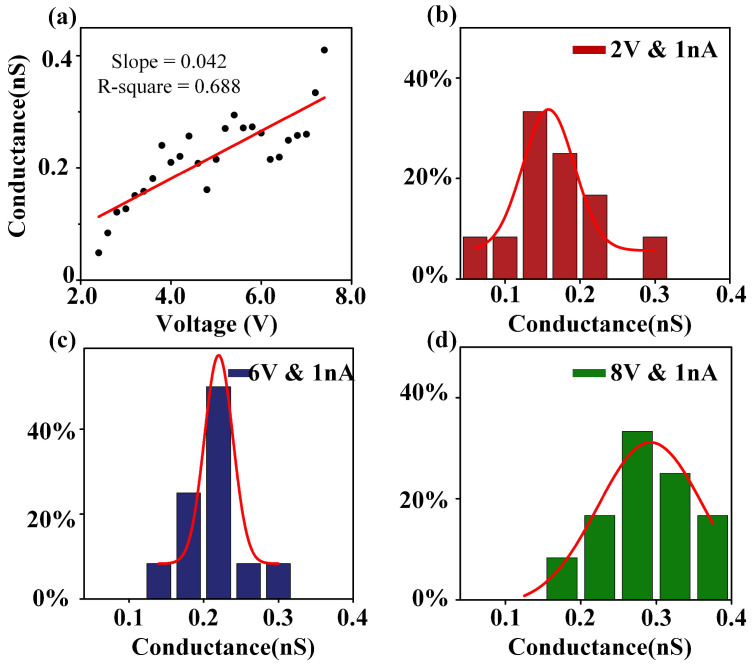
Voltage dependence of PMMA nanopore fabrication. (**a**) The nanopore conductance distribution under different voltages at a constant current (1 nA). The voltage ranges from 2.2 V to 8 V in increments of 0.2 V, resulting in an electric field range of 2.2 MV/m to 8 MV/m, significantly exceeding the breakdown electric field of the PMMA membrane. (**b**–**d**) Nanopore conductance frequency histogram (*n* = 12), fabricated with voltages of 2 V, 6 V, and 8 V. The data are fitted by Gaussian distribution, revealing average conductance values of 0.142 nS, 0.212 nS, and 0.281 nS for the respective voltages.

To construct different applied electric fields, a constant current of 1 nA was maintained while applying different voltages. In Figure 3a, the voltage started at 2.2 V and increased in increments of 0.2 V until reaching 8 V. This voltage range corresponded to an electric field range of 2.2 MV/m to 8 MV/m, which significantly exceeded the breakdown electric field of the PMMA membrane. Importantly, all these electric fields enabled the fast fabrication of PMMA nanopores. Subsequently, the conductance of the nanopore was measured using in situ IV scanning, which eliminated any influence from pipette conductance in the measurement software. It can be observed that the nanopore conductance exhibited fluctuations around the trend line under different voltages. The statistics of the linear fitting detailed in Table S2 (Supporting Materials). This result indicates that the nanopore conductance changes at a rate of 0.042 nS per 1 V increase in fabrication voltage, confirms that the strength of the electric field is a key factor affecting the initial pore size of the nanopore.

To investigate the conductance fluctuation of nanopores under the same electric field, a series of 12 repeated experiments were conducted on the same PMMA membrane using voltages of 2 V, 6 V, and 8 V. The purpose was to examine the distribution of nanopore conductance, as shown in Figure 3b–d. After performing Gaussian fitting analysis, the average conductance values corresponding to 2 V, 6 V, and 8 V were determined to be 0.142 nS, 0.212 nS, and 0.281 nS, respectively. The fluctuation percentages were calculated to be 8.075%, 4.447%, and 16.5% for each voltage, respectively. These findings indicate that the size of the nanopores fabricated in the PMMA membrane using the local high electric field method is positively correlated with the strength of the applied electric field. Moreover, the fabricated nanopores under the same electric field exhibit a normal distribution in terms of pore size. This suggests a certain degree of stability in the fabrication process, meeting the requirements for rapid and stable nanopore fabrication.

### 3.5. Current Effect for PMMA Nanopore Fabrication

In the past, researchers have primarily emphasized the significance of the voltage value in nanopore fabrication through dielectric-controlled breakdown. This voltage value plays a critical role as it directly influences the strength of the electric field utilized in the fabrication process [41,42,43]. A recent study by Vincent et al. delved into a systematic examination of the various factors that influence the breakdown and expansion of solid-state nanopores [44]. Their research suggested that the voltage parameter dictates the breakdown time, while the current setting determines the rate of expansion during the fabrication process. Understanding these dynamics is essential for controlling the fabrication process and achieving desired nanopore characteristics. In this article, given the extremely short fabrication time involved, it becomes particularly important to investigate the influence of the fabrication current.

**Figure 4:**
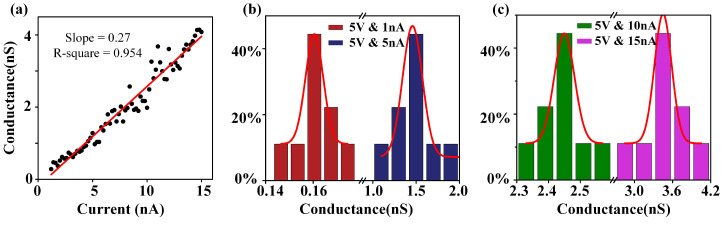
Current dependence of PMMA nanopore fabrication. (**a**) The range of currents used (1 nA to 15 nA at intervals of 0.2 nA) and the electric field strength (5 MV/m). (**b**,**c**) Nanopore conductance frequency histogram (*n* = 9), fabricated with a current of 1 nA, 5 nA,10 nA, and 15 nA, is fitted by Gaussian distribution, yielding average conductance values of 0.16 nS, 1.5 nS, 2.45 nS, and 3.5 nS, respectively.

Figure 4a depicts the relationship between nanopore conductance and the applied current value. The current starts at 1 nA and increases to 15 nA at intervals of 0.2 nA while maintaining a constant voltage of 5 V. A constant voltage of 5 V was chosen to avoid undesirable effects. If the voltage is too high, the electroosmotic flow phenomenon becomes more obvious as the current increases, leading to the gradual accumulation of the ionic solution on the surface of the PMMA membrane and increasing the contact area. In such cases, there is a risk of damaging the membrane. On the other hand, if the voltage is too low, such as 2 V, adjusting the current at this level will not result in significant changes in the conductance. Linear fitting of appiled current and nanopore conductance detailed in Table S3 (Supporting Materials). This result indicates that the nanopore conductance changes at a rate of 0.27 nS per 1 nA increase in applied current.

Furthermore, we investigated the conductance distributions of the nanopores for various currents (1, 5, 10, 15 nA) under consistent voltage conditions. Utilizing Gaussian fitting, Figure 4b,c illustrates that the central conductance values for the respective currents are 0.16 nS, 1.5 nS, 2.45 nS, and 3.5 nS. In comparison to the impact of voltage variations, it is observed that alterations in current have a more pronounced effect on nanopore conductance.

### 3.6. Characterization of PMMA Nanopores

The nanopores fabricated using positive voltages exhibit a series of asymmetric IV curves, indicating asymmetric nanopore ion-current rectification (Figure S3a, Supporting Materials). This suggests that all PMMA nanopores are tapered, with the upper part being larger than the lower part due to the application of positive voltages to the micropipette. This facilitates the use of AFM for characterization. To evaluate the diameter and morphology of the PMMA nanopores, we employed AFM to characterize a series of large-sized PMMA nanopores. They were fabricated using the same 5 V voltage and currents of 5 nA and 10 nA. The AFM images show two nanopores (Figure S3b,c, Supporting Materials), with corresponding pore diameters of 148 nm and 353 nm.

Then, further calculations were made to determine the bottom pore size of the PMMA membrane through the conductivity after breakdown. The conductance corresponding to the nanopores in the images was calculated using Equation (2) [45,46], which is consistent with the values obtained from the IV curve:

(2) $$R_{n}=\frac{\rho l}{\pi r_{t}^{2}}+\frac{\rho \cot\theta /2}{\pi }\left(\frac{1}{r_{b}}-\frac{1}{r_{t}}\right)$$

(3) $$R_{n}=\frac{\rho \cot\theta /2}{\pi r_{b}}$$

where ρ represents resistivity, which is the reciprocal of conductivity (7 S/m), *l* is the thickness of the membrane, with a value of 100 nm, $r_{b}$ and $r_{t}$ stand for the radii of the nanopore bottom and top, respectively, and θ is the cone angle, with an assumed value of 5 degrees. Typically, the radius of the nanopore is far greater than that of the bottom radius ($r_{t}>>r_{b}$). Therefore, in cases where it is not feasible to use AFM to scan the top diameter of the nanopore, we can calculate the diameter of the bottom of the nanopore by Equation (3). The bottom diameter of the nanopore is twice $r_{b}$.

By applying Equation (3), we computed the diameter of the nanopore created using 2 V & 1 nA to be approximately 0.3 nm. This calculation demonstrates that our approach is capable of fabrication sub-2 nm nanopores on the PMMA membrane.

**Figure 5:**
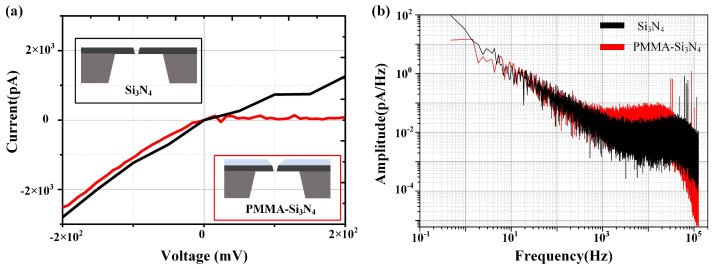
Noise of nanopores. (**a**) The IV curves of localized high electric field fast fabrication $SiN_{x}$ nanopore ($Si_{3}N_{4}$) and polymer–silicon nitride composite nanopore (PMMA-$Si_{3}N_{4}$). The fabrication parameters for the silicon nitride nanopore were 25 V & 5 nA, the nanopore size is 6.7 nm as determined from the fitted IV curve, the parameters for the fabrication polymer–silicon nitride composite nanopore were 28 V & 5 nA, and the nanopore size fitted by the IV curve is 5.2 nm. Both nanopores exhibit conical structures. (**b**) The noise performance of polymer–silicon nitride composite nanopore is significantly lower than the silicon nitride nanopore, particularly at frequencies above 30 kHz. This noise reduction can be attributed to the decreased capacitance of the nanopores after the PMMA membrane deposition.

In the end, this method of local dielectric breakdown was employed to fabrication nanopores with a size of approximately 5 nm on both the PMMA-$SiN_{x}$ composite membrane and $SiN_{x}$ membrane. The $SiN_{x}$ suspension membrane used possesses a thickness of 20 nm, and the PMMA-$SiN_{x}$ composite membrane is coated with PMMA with a thickness of 100 nm on the $SiN_{x}$. Figure 5a illustrates two variations of nanopore IV curves, whereas Figure 5b shows the noise power spectrum at a voltage of 100 mV (Axon 200B and Digidata 1550B, Molecular Devices, San Jose, CA, USA). It is evident that in the high-frequency range, the noise level of the PMMA-$SiN_{x}$ composite nanopore is lower due to its smaller capacitance.

## 4. Discussion

The voltage used in traditional dielectric breakdown methods for nanopore fabrication must be kept below the breakdown electric field of the membrane. However, when working with PMMA membranes that have a low breakdown electric field (18–22 MV/m), it becomes challenging to control the fabrication voltage due to its extremely low value. In contrast, the local high electric field approach employed in this article allows for nanopore fabrication at several (one–seven) times higher than the membrane’s breakdown electric field, proving to be feasible and effective. This high electric field method enables the direct fabrication of low-noise nanopores with a sandwich structure on materials such as $SiN_{x}$ or graphene membranes coated with PMMA [47].

Additionally, it is worth noting that the nanopore formation process using the local high electric field method is instantaneous [45]. The diameter of the nanopores is positively correlated with both voltage and current. However, we have observed that current has a greater impact on the nanopore diameter, indicating the simultaneous occurrence of intrinsic breakdown and thermal breakdown [48,49]. This finding aligns with the research conclusions of Leung et al. [44], where voltage primarily affects the process of intrinsic breakdown, while current mainly influences the process of thermal breakdown [44,50].

**Figure 6:**
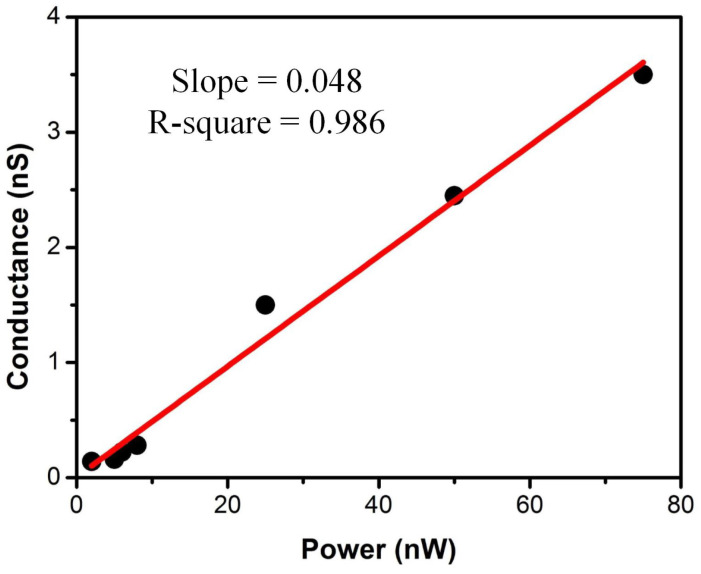
The relationship between PMMA nanopore diameter and applied power: the nanopore diameter is positively correlated with the power.

Finally, we established the relationship between fabrication parameters in Table 1 and the power with the nanopore size, as shown in Figure 6. This indicates that the nanopore size is positively correlated with the applied power. The statistics of the linear fitting detailed in Table S4 (Supporting Materials). This result indicates that the nanopore conductance changes at a rate of 0.048 nS per 1 nW increase in applied power. That can simultaneously explain the relationship between the nanopore size variation in silicon nitride and PMMA: the greater the fabrication power, the greater the energy conversion, resulting in larger nanopore sizes in the solid-state nanopore fabrication.

## 5. Conclusions

The fabrication process involved the creation of a series of polymer nanopores using local high electric field breakdown, with all breakdowns occurring instantly due to the application of an electric field that exceeded the breakdown electric field of the PMMA membrane. The results demonstrate a direct proportionality between the nanopore diameter and the fabrication voltage and current. Additionally, it was observed that the electric field intensity influences the initial pore size of the nanopores, while changes in current significantly impact the conductivity of the nanopores. Moreover, the breakdown voltage and current mechanism in the formation of nanopores was discussed, indicating that this method is capable of generating sub-10 nm nanopores. Finally, the current noise of PMMA-$SiN_{x}$ composite membrane nanopores fabricated by local dielectric breakdown was compared and analyzed in relation to $SiN_{x}$ nanopores, demonstrating the feasibility of this method for producing low-noise composite membrane nanopores.

## Figures and Tables

**Table 1:** The relationship between nanopore conductance and bottom diameter.

No.	Fabrication Parameter	Resistance (MΩ)	*r_t_* (nm)	Bottom Diameter (2*r_b_*) (nm)
1	5 V & 10 nA	408	353/2	5.20
2	5 V & 5 nA	667	148/2	3.60
3	8 V & 1 nA	3558		0.60
4	6 V & 1 nA	4504		0.46
5	5 V & 1 nA	6250		0.36
6	2 V & 1 nA	7042		0.30

## Data Availability

The data that support the findings of this study are available from the corresponding author upon reasonable request.

## Supplementary Materials

Thefollowing supporting information can be downloaded at: https://www.mdpi.com/article/10.3390/s24072109/s1.
