# Supplementary material for: Fast Fabrication Nanopores on a PMMA Membrane by a Local High Electric Field Controlled Breakdown
Source: Sensors (Basel). 2024 Mar 26;24(7):2109. doi: 10.3390/s24072109 (PMC11013984; doi:10.3390/s24072109)
Supplement: Supplementary file 1 [file sensors-24-02109-s001.zip › sensors-2913108-supplementary.pdf]

Supporting Information

Fast Fabrication Nanopores on PMMA Membrane by a Local High Electric Filed Controlled Breakdown

Shaoxi Fang[1,3#], *Delin Zeng*[2#], *Shixuan He*[1, 3], *Yadong Li*[2], *Zichen Pang*[2], *Yunjiao Wang*[1,3], *Liyuan Liang*[1,3], *Ting Weng* [1,3], *Wanyi Xie*[1,3*], *Deqiang Wang*[1,2,3*]

[1]Chongqing Key Laboratory of Multi-scale Manufacturing Technology, Chongqing Institute of Green and Intelligent Technology, Chinese Academy of Sciences, Chongqing 400714, PR China

[2]Chongqing University of Posts and Telecommunications, Chongqing 400065, PR China

[3]Chongqing School, University of Chinese Academy of Sciences, Chongqing 400714, PR China

[#]These authors contributed equally to the paper

*Corresponding Authors:

E-mails: xiewanyi@cigit.ac.cn(W. X.), dqwang@cigit.ac.cn (D.W.)

1. Preparation of PMMA membrane

This research article focuses on exploring the technique of applying nanoscale PMMA membrane onto sandwich substrates (gold, chromium, and silicon) using a fast fabrication method. The thickness of the PMMA membrane plays a crucial role in determining the fabrication electric field. In Figure S1a-d, the membrane thicknesses corresponding to different concentrations (2%, 3%, 4%, and 5%) are presented at a constant rotation speed of 3000 rpm. The measured thicknesses are 35 nm, 60 nm, 79 nm, and 100 nm, respectively. The relationship between concentration and membrane thickness exhibits linearity, as demonstrated in Figure S1e. It indicates that the membrane thickness changes at a rate of 20 nm per 1% increase in concentration.

Figures S1f-i show the preparation of PMMA membranes at varying speeds of 3000 rpm, 3500 rpm, 4000 rpm, 4500 rpm, and 5000 rpm, while maintaining a constant PMMA concentration of 2%. The corresponding membrane thicknesses measured are 35 nm, 33.25 nm, 31.87 nm, 30.6 nm, and 28.1 nm, respectively. It is evident from Figure S1g that as the rotation speed increases, there is a gradual reduction in the film thickness. However, the impact of rotation speed on thickness is considerably smaller compared to the influence of concentration.

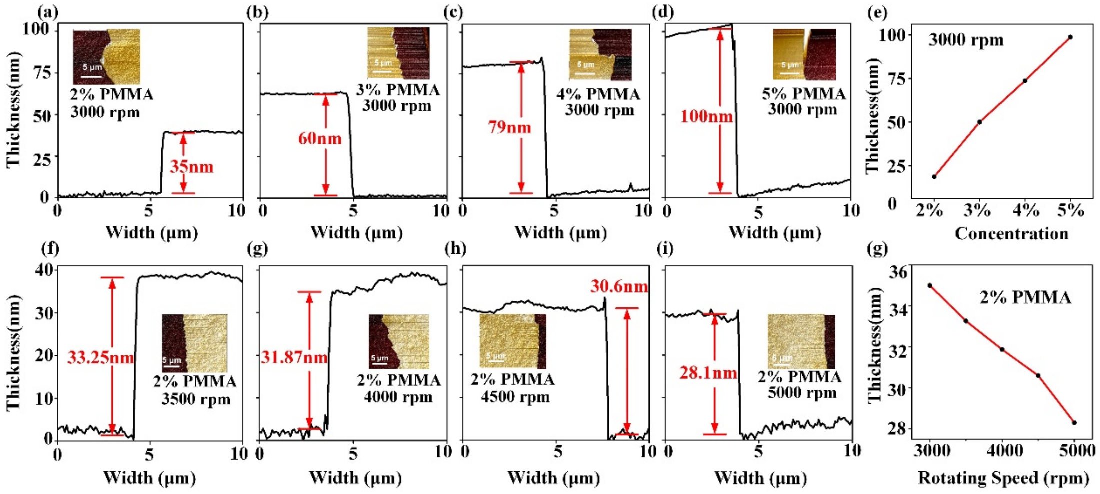

Figure S1. The PMMA membrane thicknesses is characterized based on different concentrations and rotating speeds. (a-d) AFM characterizes the thickness variations for concentrations of 2%, 3%, 4%, and 5% at a consistent rotation speed of 3000 rpm. **(e)** The line graph depicting the relationship between concentrations and the thickness of the PMMA membrane. **(f-i)** AFM characterizes the thickness measurements at various rotating speeds (3000 rpm, 3500 rpm,

4000 rpm, 4500 rpm, and 5000 rpm) while maintaining a constant PMMA concentration of 2%. **(g)** The linearity between the thickness and rotating speeds.

2. Characterization of PMMA Membrane and Nanopores

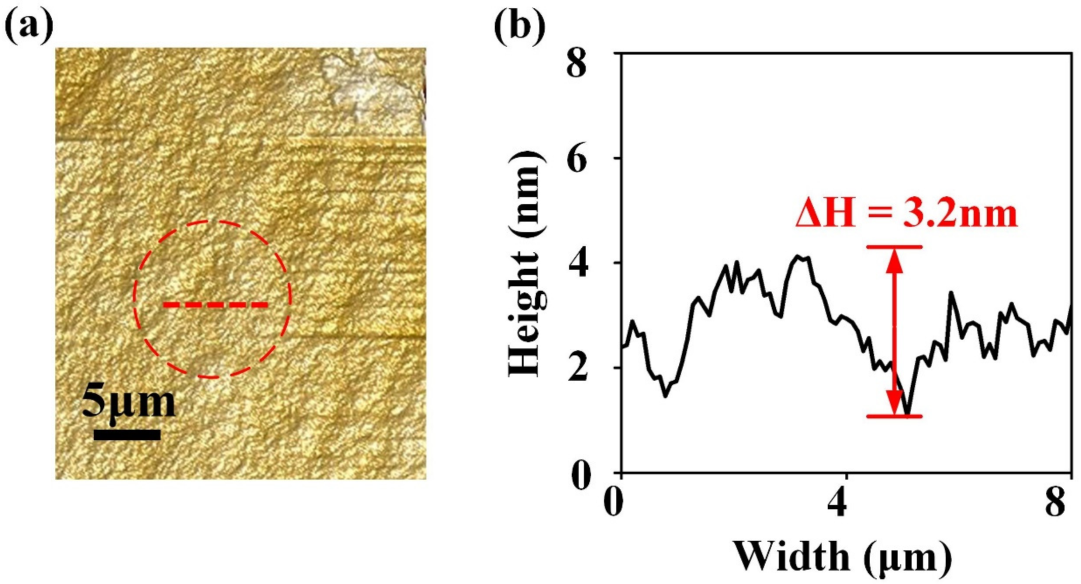

Figure S2. AFM characterizes the flatness (a, b) of PMMA membrane with a concentration of 5% at 3000 rpm.

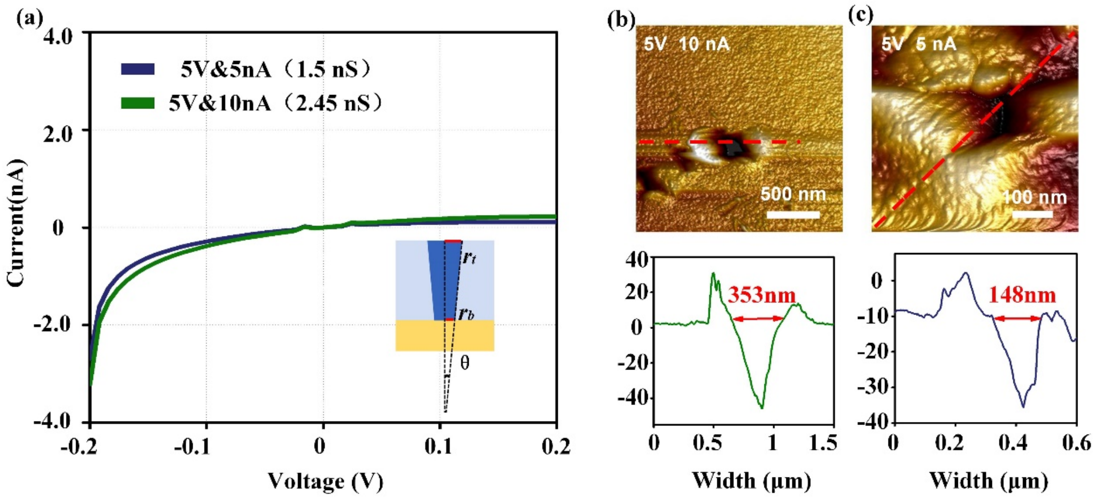

Figure S3. Characterization of PMMA nanopores. (a) The nanopore I-V curves were fabricated by different parameters (5 V&5 nA, and 5 V&10 nA). r_b and r_t are the radii of the nanopore bottom and top, respectively, and θ is the cone angle. (b, c) The nanopore images prepared with different parameters of 5 V and 10 nA (b), 5 V and 5 nA (c) have upper diameters of 353 nm and 148 nm, respectively.

3. Fabrication parameters for two types of micropipettes

Table S1. Programs of P-2000 laser-based micropipette puller

Program	HEAT	FIL	VEL	DEL	PUL	Tip Diameter

**1**	350	4	30	200	0	2.5±0.2 μm
**2**	400	4	30	200	0	1.8±0.2 μm

4. *Linear fitting of fabrication voltage and nanopore conductance*

Table S2 Summary of Linear fitting (Number of Points = 26)

	**Intercept**		**Slope**		**Statistics**
**Conductance**	Value	Standard Error	Value	Standard Error	Adj. R-Square
	0.01121	0.02898	0.04242	0.00566	0.68849

5. *Linear fitting of applied current and nanopore conductance*

Table S3 Summary of Linear fitting (Number of Points = 70)

	**Intercept**		**Slope**		**Statistics**
**Conductance**	Value	Standard Error	Value	Standard Error	Adj. R-Square
	-0.20076	0.06598	0.27738	0.00729	0.95449

6. *Linear fitting of fabrication power and nanopore conductance*

Table S4 Summary of Linear fitting

	**Intercept**		**Slope**		**Statistics**
**Conductance**	Value	Standard Error	Value	Standard Error	Adj. R-Square
	0.00763	0.08234	0.04796	0.00231	0.98622
